# Nutritional Value and Food Safety Assessment of Single-Cell Protein Derived from *Ralstonia eutropha* for Food Applications

**DOI:** 10.3390/foods15101813

**Published:** 2026-05-20

**Authors:** Xiaoyan You, Le Zhang, Ling Chen, Hui Wang, Hong Zou, Zhiguang Zhu, Guoping Zhao

**Affiliations:** 1 Henan Engineering Research Center of Food Microbiology, College of Food and Bioengineering, Henan University of Science and Technology, Luoyang 471023, China; zl18300727515@163.com; 2 National Center of Technology Innovation for Synthetic Biology, Tianjin Institute of Industrial Biotechnology, Chinese Academy of Sciences (CAS), Tianjin 300308, China; wanghuih@tju.edu.cn (H.W.); zhu_zg@tib.cas.cn (Z.Z.); 3 Haihe Laboratory of Synthetic Biology, Tianjin 300308, China; 4 Guangdong Detection Center of Microbiology, Institute of Microbiology, Guangdong Academy of Sciences, Guangzhou 510070, China; chenling@gdim.cn; 5 School of Life Sciences, Faculty of Medicine, Tianjin Key Laboratory of Function and Application of Biological Macromolecular Structures, Tianjin University, Tianjin 300072, China; 6 Engineering Laboratory for Nutrition, Shanghai Institute of Nutrition and Health, Chinese Academy of Sciences, Shanghai 200031, China; hzhou01@sinh.ac.cn; 7 CAS-Key Laboratory of Synthetic Biology, CAS Center for Excellence in Molecular Plant Sciences, Institute of Plant Physiology and Ecology, Chinese Academy of Sciences, Shanghai 200032, China

**Keywords:** *Ralstonia eutropha* H16, single-cell protein, nutritional evaluation, safety assessment, protein quality, sustainable protein

## Abstract

The growing global protein demand and environmental concerns from conventional animal agriculture have driven the exploration of sustainable alternative protein sources. Single-cell proteins (SCPs) from microbial fermentation offer a promising solution. This study comprehensively evaluated the nutritional value and safety profile of SCP produced from *Ralstonia eutropha* H16 through integrated *in vitro* and *in vivo* assessments. Nutritional analyses revealed a high crude protein content of 71.87 ± 5.05 g/100 g dry weight, with total amino acids of 53.67 ± 1.05 g/100 g. The essential amino acid content was 24.38 ± 0.51 g/100 g, accounting for 45% of the total amino acids. An essential amino acid index (EAAI) of 1.46 ± 0.04 and an amino acid score (AAS) of 0.83 ± 0.06 confirmed its classification as a high-quality protein source according to FAO/WHO standards. *In vivo* rat feeding trials demonstrated an adjusted protein efficiency ratio (PER) of 1.81, exceeding common plant proteins such as wheat (0.8–1.1). True digestibility (TD) reached 85.73%, with a biological value (BV) of 49.37%, net protein utilization (NPU) of 42.33%, and protein digestibility-corrected amino acid score (PDCAAS) of 0.71. Comprehensive safety assessments included chemical contaminant screening, acute oral toxicity studies in rats and mice, *in vitro* chromosome aberration tests, and erythrocyte micronucleus tests. Heavy metals and aflatoxin B_1_ levels were below regulatory limits. Acute oral toxicity studies established LD_50_ values exceeding 10,000 mg/kg body weight in both rodent species, classifying this protein source as practically non-toxic. The 28-day sub-acute toxicity study showed no significant adverse effects at low doses (6.25% protein replacement). Both genotoxicity assays (mammalian cell chromosome aberration assay and mammalian erythrocyte micronucleus test) returned negative results. These findings establish *R. eutropha* H16-derived SCP as a safe, nutritious, and sustainable protein source with considerable potential for feed and food applications, contributing to global food security and environmental sustainability.

## 1. Introduction

The global population is projected to reach 10 billion by 2050, driving protein demand to an estimated 1.25 billion tons annually [1]. Conventional animal protein production has become increasingly unsustainable, contributing approximately 14.5% of global greenhouse gas emissions, occupying 77% of agricultural land while providing only 18% of global calories, and consuming vast freshwater resources [2,3]. Beyond environmental concerns, industrial livestock farming raises significant animal welfare issues, with over 56 billion land animals slaughtered annually for food production [4]. This confluence of environmental degradation, resource scarcity, and ethical considerations necessitates a fundamental shift toward alternative protein sources that can deliver nutritional adequacy while minimizing ecological footprint and eliminating animal suffering [5,6,7].

Single-cell proteins (SCPs), derived from microbial biomass including bacteria, yeasts, fungi, and microalgae, have emerged as a promising solution to address global protein security [8]. SCP offers multiple advantages: (1) exceptionally high protein content in the bacterial SCP (50–80% dry weight) [9,10]; (2) rapid growth rates enabling high production efficiency [11]; (3) minimal land and water requirements compared to conventional agriculture [12]; (4) ability to utilize diverse substrates, including industrial and agricultural waste streams [13]; (5) balanced amino acid composition comparable to conventional protein sources [14]. Despite these advantages, the commercial success of SCP depends critically on the selection of optimal microbial chassis with superior performance characteristics and the rigorous validation of both nutritional value and safety profiles.

Among candidate microorganisms for SCP production, *Ralstonia eutropha* (also known as *Cupriavidus necator*), a facultative chemolithoautotrophic Gram-negative bacterium, has garnered increasing attention due to its exceptional properties [15]. First, *R. eutropha* demonstrates remarkable growth capacity and biomass accumulation efficiency, with protein content reaching up to 69–83% [16]. Second, the bacterium exhibits extraordinary metabolic versatility [17] and is capable of utilizing diverse carbon and nitrogen sources, including volatile fatty acids [18], lignocellulosic derivatives [19], and even CO_2_ via chemolithoautotrophic pathways [20], thereby offering opportunities for “grain-free” production and waste valorization. Third, unlike many industrial strains, *R. eutropha* H16 is non-pathogenic and possesses Generally Recognized as Safe (GRAS) potential, a crucial prerequisite for food and feed applications [21]. Fourth, its well-characterized genome and amenability to genetic engineering enable precision optimization for enhanced protein yield and quality [17,21]. These inherent advantages of *R. eutropha* facilitate flexible adaptation to diverse cultivation scenarios, positioning it as an ideal SCP production strain.

While pioneering studies explored hydrogenotrophic bacteria as protein sources, including early investigations of *Hydrogenomonas eutropha* (the former nomenclature of *R. eutropha*) [21], these studies were limited in scope and analytical rigor by contemporary methodological constraints. Recent research has primarily focused on genetic engineering and metabolic pathway optimization, with limited systematic evaluation of the nutritional properties and safety profiles essential for regulatory approval and consumer acceptance. Greife et al. (1980) [22] examined nitrogen utilization in broilers fed *Alcaligenes eutrophus*, reporting normal growth but reduced apparent nitrogen digestibility above 10% inclusion. Shapira and Mandel (1968) and Calloway and Kumar (1969) found that *H. eutropha* exhibited amino acid patterns comparable to casein, though with slightly lower biological value attributed to sulfur amino acid deficiency [23,24]. More recently, Modica et al. (2023) [25] conducted genotoxicity and repeated-dose oral toxicity studies on PHB-deficient *Cupriavidus necator*, finding no adverse effects.

Although *R. eutropha* demonstrates remarkable performance in SCP production, its qualification as a legitimate food ingredient fundamentally depends on ensuring absolute safety and reliable nutritional quality. Achieving the transition from “producible” to “edible” necessitates systematic resolution of nutritional and safety assessment challenges. The reasons are as follows: First, *Ralstonia eutropha* H16 is not yet listed among strains permitted for food use by China’s National Health Commission; its derived SCP is therefore classified as a novel food ingredient requiring rigorous pre-market safety evaluation. Second, existing food safety frameworks were designed for conventional materials and offer limited guidance for proteins produced via non-conventional microbial processes. This study aims to systematically assess the nutritional value and food safety of novel SCP derived from *R. eutropha* through a more comprehensive approach integrating *in vivo* and *in vitro* perspectives. Specifically, at the nutritional level, we will precisely analyze protein and amino acid composition (particularly essential amino acid patterns and content), and evaluate protein efficiency ratio and protein digestibility through *in vivo* animal experiments to preliminarily predict nutritional value. At the safety level, we will construct a hierarchical evaluation framework: initially conducting *in vitro* assessments to rapidly identify potential risks and subsequently performing standardized *in vivo* animal experiments to comprehensively observe overall responses, including mental status and mortality following protein ingestion, thereby evaluating long-term consumption safety. This integrated *in vivo* and *in vitro* evaluation approach not only overcomes the limitations of single-model assessments and enhances the scientific validity and reliability of evaluation outcomes but also provides deeper insights into the metabolic fate and biological effects of *R. eutropha* proteins within living organisms. By providing regulatory-grade evidence for both nutritional quality and biosafety, this research establishes the scientific foundation necessary for regulatory approval and commercial deployment of *R. eutropha* H16 SCP. Ultimately, this study provides the evidence base required for industry adoption and consumer acceptance, positioning *R. eutropha* H16-derived SCP as a viable component of the transition toward environmentally responsible and ethically sound protein production systems.

## 2. Materials and Methods

### 2.1. Single-Cell Protein Preparation

*Ralstonia eutropha* H16 strain was provided by Prof. Zhiguang Zhu (Tianjin Institute of Industrial Biotechnology, Chinese Academy of Sciences, Tianjin, China). The strain was routinely cultivated in Luria–Bertani (LB) broth supplemented with gentamicin sulfate at a final concentration of 10 µg/mL. Prior to experimental procedures, single colonies were isolated on LB agar plates and subcultured twice in fresh medium to ensure culture purity and physiological consistency. For biomass production, the strain was cultivated in an inorganic salt medium containing the following components (per liter): Na_2_HPO_4_·12H_2_O 9 g; KH_2_PO_4_ 1.5 g; ferric ammonium citrate 0.005 g; CaCl_2_·2H_2_O 0.02 g; NaHCO_3_ 0.5 g; MgSO_4_·7H_2_O 0.2 g; fructose 10 g; and 1 mL of trace element solution. The trace element solution was composed of (per liter) the following: CuSO_4_·5H_2_O 10 mg; MnCl_2_·4H_2_O 30 mg; ZnSO_4_·7H_2_O 100 mg; CoCl_2_·6H_2_O 200 mg; NiCl_2_·6H_2_O 20 mg; Na_2_MoO_4_·2H_2_O 30 mg; and boric acid 300 mg. Large-scale cultivation of *R. eutropha* H16 was performed in a 1000 L bioreactor under optimized conditions (30 °C, pH 7.0, 200 rpm, 20% dissolved oxygen). After fermentation (72 h), the cell culture was harvested and concentrated using ceramic membrane filtration. Following concentration, the concentrate was washed three times to remove components from the culture medium. The concentrated cell suspension was disrupted using a homogenizer, yielding a disrupted cell lysate. The homogenizer operating pressure was 1000 bar. Spray drying of the cell lysate yielded SCP powder. The prepared SCP was stored in sealed containers at −80 °C and used for subsequent nutritional and safety analyses.

### 2.2. In Vitro Nutritional Evaluation

#### Nutritional Composition Analysis and Amino Acid Scoring

Composition of *R. eutropha* H16 SCP was determined according to Chinese National Food Safety Standards: moisture content by oven-drying method (GB 5009.3-2016) [26]; ash content by high-temperature muffle furnace incineration (GB 5009.4-2016) [27]; crude protein by Kjeldahl method with conversion factor N × 6.25 (GB 5009.5-2016) [28]; crude fat by Soxhlet extraction with diethyl ether (GB/T 5009.6-2016) [29]; and dietary fiber by the enzymatic-gravimetric method (GB/T 5009.88-2016) [30]. Carbohydrate content was calculated by the difference method. All analyses were performed in triplicate.

Amino acid profiles were analyzed by high-performance liquid chromatography (HPLC) following acid hydrolysis (GB/T 5009.124-2016) [31], except for tryptophan, which was excluded due to its destruction during hydrolysis. Amino acid composition was expressed as g/100 g protein and compared with the FAO/WHO reference pattern for adults [32]. The ratio of essential amino acids to total amino acids (E/T, %) was calculated. The amino acid score (AAS) and essential amino acid index (EAAI) were determined according to established methods [33]:

(1) AAS(Amino acid score) = m/n

(2) $$EAAI=\sqrt[{n}]{\left[\left(a_{1}/a_{r1}\right)\times \left(a_{2}/a_{r2}\right)\times \ldots \times \left(a_{n}/a_{rn}\right)\right]}$$

where m represents the content of limiting amino acids in the test protein, n represents the content of the same amino acid in the reference protein, a represents essential amino acid content in the test protein, aᵣ represents content in the reference protein (egg), and n is the number of essential amino acids evaluated.

### 2.3. In Vivo Nutritional Evaluation

#### 2.3.1. Protein Efficiency Ratio (PER) Study

##### Experimental Diets, Animal Housing, and Husbandry

Two isocaloric, isonitrogenous diets (10% protein) were formulated according to AIN-93G specifications (Table S1): (1) *R. eutropha* H16 SCP test diet containing 148.20 g/kg bacterial protein powder; and (2) casein control diet containing 104.00 g/kg casein plus 1.50 g/kg L-cystine. Both diets were supplemented with corn starch, maltodextrin, sucrose, cellulose, soybean oil, mineral mix (AIN-93G-MX), vitamin mix (AIN-93-VX), choline bitartrate, and tert-butylhydroquinone (TBHQ) antioxidant. Forty weanling Sprague Dawley (SD) rats (3 weeks old, body weight 70–80 g, obtained from Shanghai SLAC Laboratory Animal Co., Ltd., Shanghai, China) were housed individually in steel cages under controlled environmental conditions (21 ± 2 °C, 55 ± 20% relative humidity, 10–15 air changes/hour, 12:12 h light: dark cycle). After a 4-day acclimatization period on standard laboratory chow, rats were randomly assigned to two groups (n = 20/group, equal sex distribution) and fed their respective experimental diets ad libitum for 28 days with free access to deionized water. All procedures were approved by the Shanghai Institute of Nutrition and Health, Chinese Academy of Sciences, Institutional Animal Care and Use Committee (Approval No.: SINH-2024-LY-2).

##### Growth Performance and PER Determination

Body weight was recorded weekly, and food intake was monitored every 2–3 days throughout the 28-day experimental period. Weight gain (g) and cumulative protein intake (g) were calculated for each rat. The food efficiency ratio (FER) [34] and protein efficiency ratio (PER) [35] were calculated as

(3)  FER=Weight gain(g)/Total food intake(g)

(4)  PER=Weight gain(g)/Protein intake(g)

The PER values were adjusted relative to a standard casein control (assumed PER = 2.5) to enable comparison with literature values:

(5)  Adjusted PER =(Test PER/Casein PER)×2.5

#### 2.3.2. Protein Digestibility and Biological Value Study

##### Animals and Experimental Design

Two diets were prepared: (1) *R. eutropha* H16 SCP test diet (10% protein, composition identical to Section Experimental Diets, Animal Housing, and Husbandry; and (2) protein-free (non-protein) control diet with protein source replaced by equivalent corn starch to maintain isocaloric content (Table S2). Eight SD rats (3 weeks old, body weight 110–130 g, equal sex distribution) from Shanghai SLAC Laboratory Animal Co., Ltd. were housed individually in metabolic cages designed for the quantitative collection of feces and urine. After 7 days of acclimatization on AIN-93G standard diet, the rats were randomly assigned to two groups (n = 4/group): (1) SCP test group; (2) protein-free control group. Rats were fed their respective diets ad libitum for 9 days with free access to water under controlled environmental conditions (as described in Section Experimental Diets, Animal Housing, and Husbandry. Animal procedures were approved by the Shanghai Institute of Nutrition and Health, Chinese Academy of Sciences, Institutional Animal Care and Use Committee (Approval No.: SINH-2024-LY-2).

##### Calculation of *R. eutropha* H16 SCP Bioavailability

During the 5-day experimental period (Days 5–9), daily measurements were recorded for body weight, food intake, water consumption, and quantitative collection of feces and urine. Fecal samples were oven-dried (105 °C) to constant weight, ground, and stored at −20 °C. Urine samples were collected daily over 5% HCl to prevent nitrogen loss, measured volumetrically, and stored at −20 °C. Total nitrogen content in feed, feces, and urine was determined by the Kjeldahl method [36]. True digestibility (TD), biological value (BV), net protein utilization (NPU), and the protein digestibility-corrected amino acid score (PDCAAS) were calculated according to the method of Berrazaga et al. [37,38].

### 2.4. In Vitro Safety Evaluation

#### 2.4.1. Chemical Contaminant Screening and Microbiological Safety Assessment

Total arsenic (As) was determined by dry ashing followed by hydride generation-atomic fluorescence spectrometry (HG-AFS) with minor modifications [39]. Approximately 0.5 g of homogenized dry sample was taken, mixed with about 2.5 mL of ashing aid suspension and 5 mL of nitric acid, and evaporated to dryness on a hot plate, followed by mineralization. The ash was dissolved in hydrochloric acid, allowed to stand for approximately 30 min, and then diluted to volume with hydrochloric acid. Lead (Pb), cadmium (Cd), and chromium (Cr) were analyzed using flame atomic absorption spectrometry (FAAS) after dry ashing, while mercury (Hg) was measured by microwave digestion–atomic fluorescence spectrometry (MD-AFS), with sample digestion carried out in closed vessels using a hydrochloric–nitric acid mixture as the digestion medium [40,41]. The resulting ash was dissolved in 1 N nitric acid, and then, lead and cadmium were extracted with methyl isobutyl ketone (MIBK) after complexation of the metals with ammonium 1-pyrrolidinedithiocarbamate (APDC). Fluorine (F) was quantified using an ion-selective electrode (ISE) [42]. Then, 25 mL of total ionic strength adjustment buffer was added, and the mixture was diluted to the mark with distilled water. The fluoride ion electrode and the calomel electrode were connected to the positive and negative terminals of the measuring instrument. Aflatoxin B_1_ (AFB_1_) was analyzed via liquid chromatography–tandem mass spectrometry (LC-MS/MS) [43]. Microbiological analyses were performed according to Sanjee et al. [44]. The total bacterial count was enumerated using the standard plate count method; coliform bacteria were detected via lauryl sulfate tryptose (LST) broth fermentation; *Salmonella* was investigated through cultural isolation followed by biochemical identification. All analyses were conducted in triplicate under aseptic conditions.

#### 2.4.2. Mammalian Chromosome Aberration Test

The *in vitro* mammalian cell chromosome aberration test was performed on the Chinese hamster lung (CHL) cell line (CL-0060) according to GB 15193.23-2014 guidelines [45]. Based on preliminary cytotoxicity assays showing no toxicity at 5 mg/mL, the test item concentrations were set at 1.25, 2.5, and 5 mg/mL (with and without S9 metabolic activation). DMEM served as the negative control. Positive controls included methyl methanesulfonate (MMS, 50 µg/mL; Macklin, C14265301) for the non-activated (−S9) condition and cyclophosphamide (CPA, 10 µg/mL; Aladdin, A2112310) for the S9-activated (+S9) condition. CHL cells (1 × 10^6^) were seeded into 25 cm^2^ flasks and incubated for 18–24 h to reach approximately 85% subconfluence. For treatment, the medium was replaced with a serum-free medium containing the specified test item concentrations, either with or without the S9 mix. After incubation for 2–6 h, the treatment medium was removed. The cells were washed thrice with PBS, replenished with fresh medium containing 10% fetal bovine serum (FBS), and incubated for a total of 24 h post-treatment. To arrest cells at metaphase, colchicine (0.4 µg/mL final concentration) was added 2–4 h prior to cellular harvest. Cells were then detached using 0.25% trypsin, collected, and stained with 10% Giemsa solution for 15 min for morphological slide preparation. Slides were examined under an oil-immersion microscope. For cells exhibiting chromosomal aberrations, the microscopic field coordinates and aberration types were recorded.

### 2.5. In Vivo Safety Evaluation

#### 2.5.1. Mammalian Erythrocyte Micronucleus Test

The *in vivo* micronucleus assay was conducted in accordance with GB 15193.5-2014 to assess potential genotoxicity [46]. Fifty Kunming (KM) mice (6–8 weeks old, body weight 25–30 g, equal sex distribution) from Guangdong Province Medical Laboratory Animal Center were randomly allocated to five groups (n = 10/group): three SCP dose groups (2.5, 5.0, 10.0 g/kg BW), one negative control (distilled water), and one positive control (cyclophosphamide, CP, 0.04 g/kg BW). Test substances were administered by gavage (20 mL/kg volume) at 0 and 24 h (30 h protocol). Six hours after the second administration, mice were euthanized by cervical dislocation. Bone marrow cells from both femurs were flushed with fetal bovine serum, smeared on glass slides, air-dried, fixed with methanol, and stained with Giemsa solution. For each animal, 1000 polychromatic erythrocytes (PCE) were examined microscopically for micronucleus frequency at 1000× magnification, and the PCE/normochromatic erythrocyte (NCE) ratio was determined by scoring 200 total erythrocytes. All procedures received ethical approval from the Institute of Microbiology, Guangdong Academy of Sciences, Laboratory Animal Management and Ethics Committee (Approval No.: GT-IACUC202411071).

#### 2.5.2. Acute Oral Toxicity Study

Acute oral toxicity of *R. eutropha* H16 SCP was evaluated in both mice and rats in accordance with the Chinese National Standard GB 15193.3-2014 [47]. Thirty KM mice (6–8 weeks old; body weight: 25–28 g) were obtained from the Guangdong Province Medical Laboratory Animal Center and randomly assigned to a test group (n = 20) and a control group (n = 10). Forty SPF-grade SD rats (6–8 weeks old; body weight: 160–210 g; equal sex distribution) were randomly allocated to test and control groups (n = 20/group) and housed under the conditions described in Section Experimental Diets, Animal Housing, and Husbandry. Following a fasting period (4–6 h for mice; approximately 16 h for rats) with free access to water, animals received a single oral gavage dose of *R. eutropha* H16 SCP at 10,231.3 mg/kg BW (20 mL/kg volume) for mice or 10,171.1 mg/kg BW (10 mL/kg volume) for rats; control animals received an equivalent volume of water vehicle. All animals were monitored continuously for the first 4 h post-dosing and twice daily thereafter for clinical signs of toxicity, morbidity, and mortality over a 14-day observation period. Body weight was recorded on Days 0, 7, and 14 for mice and weekly for rats. The median lethal dose (LD_50_) was estimated from mortality data. All procedures were approved by the Laboratory Animal Management and Ethics Committee of the Institute of Microbiology, Guangdong Academy of Sciences (Approval Nos.: GT-IACUC202411071 and GT-IACUC202504242).

#### 2.5.3. Subacute Toxicity Study

A subacute oral toxicity study was conducted in Sprague Dawley rats in accordance with the Chinese National Standard GB/T 15193.22-2014 [48]. Eighty SPF-grade SD rats (body weight: 70–140 g; age: 6–8 weeks) were stratified by body weight and randomly allocated to four groups (n = 10/sex/group): a control group receiving the basal diet, and three dose groups in which 6.25% (low-dose), 12.5% (medium-dose), and 25% (high-dose) of dietary protein was replaced by *R. eutropha* H16 SCP. All groups had ad libitum access to their respective diets for 28 consecutive days. Body weight and food consumption were recorded weekly. Cage-side observations were performed every other day to document any clinical signs of toxicity. Urine was collected 1–2 days prior to scheduled sacrifice. On the day of necropsy, blood was drawn into anticoagulant tubes. Whole-blood samples were analyzed for hematological parameters on the day of collection; plasma and urine aliquots were stored at −80 °C for subsequent clinical chemistry and urinalysis, respectively. A comprehensive gross necropsy was performed on all animals. Absolute organ weights and organ-to-body weight ratios were determined for the heart, thymus, adrenal glands, liver, kidneys, spleen, and testes/ovaries. Tissue samples from the brain, thyroid, thymus, heart, liver, spleen, kidneys, adrenal glands, stomach, duodenum, colon, pancreas, mesenteric lymph nodes, testes/ovaries, and urinary bladder were collected and preserved in fixative for microscopic examination. Tissues exhibiting gross abnormalities were further subjected to histopathological evaluation. All animal procedures were reviewed and approved by the Institutional Animal Care and Use Committee of the Shanghai Institute of Nutrition and Health, Chinese Academy of Sciences (Approval No.: SINH-2024-LY-2).

### 2.6. Statistical Analysis

Data are presented as mean ± standard deviation (SD) with a sample size of n ≥ 3 for chemical analyses and n ≥ 5 for animal studies. Statistical comparisons between groups were performed using one-way analysis of variance (ANOVA) followed by Tukey’s post hoc test or Student’s *t*-test as appropriate (SPSS 27.0, IBM Corp., Armonk, NY, USA). Graphical representations were generated using GraphPad Prism 9.5.0 (GraphPad Software, San Diego, CA, USA). Statistical significance was defined as *p* < 0.05 (*), *p* < 0.01 (**), or *p* < 0.001 (***).

## 3. Results

### 3.1. Results of Single-Cell Protein Preparation

One liter of *R. eutropha* H16 seed culture (OD 5.5) was aseptically inoculated into a 100 L fermentation seed tank (fermentation broth 60 L) and cultured for 12 h, achieving an OD of 5.39. Thirty liters of seed culture was inoculated into two 1000 L fermentation tanks containing 750 L and 700 L of fermentation broth, respectively. The fructose solution was fed at 3 kg/h (fructose dissolved 1:1 in water). Fermentation was conducted for 72 h under the following conditions: 30 °C, 200 rpm agitation, and pH 7. After fermentation (final OD_600_ 54.8), 700 L of bacterial broth was collected and concentrated using ceramic membrane filtration. The concentrate was then washed three times to remove medium components, yielding approximately 140 L of concentrated bacterial broth. One hundred thirty-five liters of concentrated bacterial broth was disrupted using a homogenizer at a working pressure of 1000 bar, yielding approximately 135 L of cell suspension. The bacterial suspension was processed by spray drying to obtain 9321 g of powder.

### 3.2. Nutritional Composition Analysis and Amino Acid Scoring

Nutritional composition analysis revealed that *R. eutropha* H16-derived SCP exhibited an exceptionally high crude protein content of 71.87 ± 5.05 g/100 g dry weight, substantially exceeding the values previously reported for other microbial proteins, such as *Chlorella pyrenoidosa*, *Rhodopseudomonas palustris*, *Saccharomyces cerevisiae*, and *Candida utilis* (Table S3). The SCP contained minimal lipid (2.00 ± 2.17 g/100 g), low moisture (2.48 ± 1.38 g/100 g), and moderate ash (4.87 ± 0.95 g/100 g). Dietary fiber was not detected. These compositional characteristics position *R. eutropha* H16 SCP as an ultra-high-protein low-fat ingredient suitable for protein fortification applications.

Comprehensive amino acid analyses (excluding tryptophan and cysteine) revealed a total amino acid content of 53.67 ± 1.05 g/100 g in *R. eutropha* H16 SCP (Table 1). Glutamic acid was the most abundant amino acid (7.55 ± 0.51 g/100 g), followed by alanine (6.00 ± 0.07 g/100 g) and leucine (5.12 ± 0.17 g/100 g). Among essential amino acids, leucine content was the highest (5.12 ± 0.17 g/100 g), while histidine was the lowest (1.25 ± 0.10 g/100 g). The methionine content was 1.37 ± 0.09 g/100 g, and aromatic amino acids phenylalanine and tyrosine summed to 5.29 ± 0.67 g/100 g. The essential amino acid content (∑EAA = 24.38 ± 0.51 g/100 g) represented 45% of total amino acids (∑EAA/∑TAA = 0.45 ± 0.01), exceeding the 36% threshold for ideal protein quality [32]. The ratio of essential to non-essential amino acids (∑EAA/∑NEAA) was 0.83 ± 0.02, comparable to milk (0.78) and superior to wheat (0.37).

The amino acid scores are presented in Table 2. The analysis identified histidine as the first limiting amino acid in the single-cell protein derived from *R. eutropha* H16. The essential amino acid index (EAAI = 1.46 ± 0.04) significantly exceeded the threshold for high-quality proteins (EAAI ≥ 0.95), indicating superior nutritional value. Notably, *R. eutropha* H16 SCP surpassed plant-based proteins, including soybean (∑EAA = 19.90 g/100 g), corn (∑EAA = 21.00 g/100 g), and wheat (∑EAA = 18.00 g/100 g), in essential amino acid content, and it was comparable to that of casein (24.80 g/100 g), though slightly below whey (34.10 g/100 g) and milk (30.30 g/100 g).

### 3.3. Bioavailability of R. eutropha H16 SCP

#### 3.3.1. Protein Efficiency Ratio (PER)

**Figure 1:**
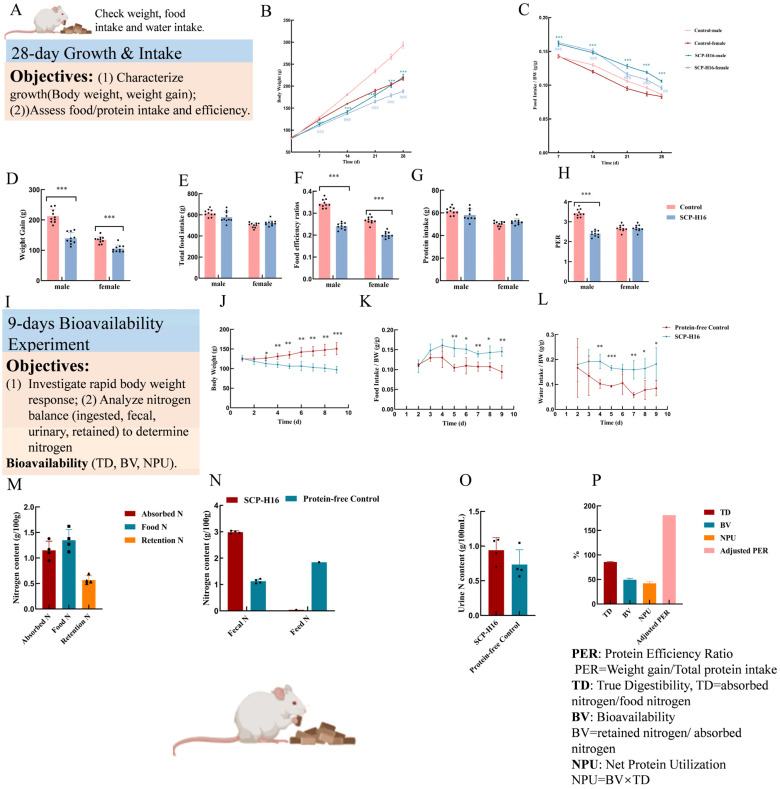
*In vitro* nutritional evaluation. (**A**) *In vitro* experiment design. (**B**) The weight change over 28 days. (**C**) Changes in food intake over 28 days, *** extremely significantly different from the control group in the male test, *p* < 0.001; ## highly significantly different from the control group in the female test, *p* < 0.01; ### extremely significantly different from the control group in the female test, *p* < 0.001. (**D**) Weight gain during the 28-day feeding trial, n = 10, *** extremely significantly different from the control group, *p* < 0.001. (**E**) Total food intake over 28 days. (**F**) Food efficiency ratio, n = 10, *** extremely significantly different from the control group, *p* < 0.001. (**G**) Total protein intake over 28 days. (**H**) Protein efficiency ratio, *** extremely significantly different from the control group, *p* < 0.001. (**I**) 9-days bioavailability experiment. (**J**) The weight change over 9 days. (**K**) Changes in food intake over 9 days. (**L**) Changes in water intake over 9 days. * Significantly different from the protein-free control group, *p* < 0.05; ** highly significantly different from the protein-free control group, *p* < 0.01; *** extremely significantly different from the protein-free control group, *p* < 0.001. (**M**) Nitrogen content in absorbed, feed, and retained fractions. (**N**) Urinary nitrogen content in different groups. (**O**) The content of fecal nitrogen and feed in different groups, where endogenous fecal N = fecal N of protein-free control group; absorbed N = feed N − (fecal N − endogenous fecal N); and endogenous urinary N = urinary N of protein-free control group. (**P**) Bioavailability of single-cell protein from *R. eutropha* H16 in protein bioavailability experiment: PER: protein efficiency ratio; TD: true digestibility; BV: biological value; NPU: net protein utilization.

At the initiation of the 28-day feeding trial, no significant differences in mean body weight were observed between the SCP test and casein control groups for either sex (male control: 80.68 ± 4.16 g; male test: 80.65 ± 3.61 g; female control: 82.60 ± 2.01 g; female test: 82.65 ± 2.50 g; *p* > 0.05) (Figure 1B). Following dietary intervention, all groups exhibited weight gain, though the magnitude differed significantly between treatments. At Day 28, final body weights were significantly lower in SCP groups compared to casein controls (*p* < 0.001): male control (293.34 ± 21.66 g) vs. male test (220.27 ± 20.15 g); female control (217.12 ± 11.40 g) vs. female test (188.20 ± 11.46 g) (Figure 1B). Throughout the experimental period, the food intake of the SCP group was significantly higher than that of the control group (*p* < 0.01 to *p* < 0.001) (Figure 1C). Consequently, the total weight gain was highly significantly reduced in SCP-fed rats: male controls gained 212.66 ± 22.36 g versus 139.63 ± 19.14 g for the male test (*p* < 0.001); female controls gained 134.52 ± 11.31 g versus 105.56 ± 12.16 g for the female test (*p* < 0.001) (Figure 1D).

The food efficiency ratio, an indicator of feed conversion efficiency, differed significantly between dietary groups (Figure 1F). In male rats, FER was 0.24 ± 0.01 for the SCP group compared to 0.34 ± 0.02 for the casein control (*p* < 0.001). Female rats exhibited FER values of 0.20 ± 0.01 (SCP) versus 0.27 ± 0.02 (casein) (*p* > 0.001). These results indicate that SCP-fed rats required greater food intake per unit body weight gain, suggesting lower feed conversion efficiency relative to casein. This may be because casein tends to coagulate in the stomach, potentially delaying gastric emptying and reducing short-term feeding desire [51]. The protein efficiency ratio, a standard metric for protein quality assessment, revealed significant differences between dietary proteins (Figure 1H). Over the 28-day trial, male rats fed *R. eutropha* H16 SCP achieved a PER of 2.41 ± 0.05, significantly lower than the casein control PER of 3.44 ± 0.06 (*p* < 0.001). Female SCP-fed rats exhibited a PER of 2.01 ± 0.04 compared to 2.68 ± 0.05 for casein controls. After standardizing casein PER to the reference value of 2.5, adjusted PER values for *R. eutropha* H16 SCP were 1.75 (male) and 1.88 (female). Comparative analysis with literature-reported PER values demonstrated that *R. eutropha* H16 SCP (Adjusted PER =1.81) exceeded plant-based proteins, including wheat (PER = 0.8), while remaining inferior to high-quality animal proteins such as casein, egg, and whey (Figure S1).

#### 3.3.2. Protein Digestibility and Utilization Indices

Rats fed the *R. eutropha* H16 SCP diet exhibited significantly higher body weight throughout the 9-day experimental period compared to the protein-free control group (Figure 1J). Body weight differences became statistically significant from Day 3 onward (*p* < 0.05), with divergence increasing progressively through Day 9 (*p* < 0.001). Over the experimental period, SCP-fed rats gained 20.69% body weight, whereas protein-free controls lost 22.92% body weight, underscoring the essential role of dietary protein in growth maintenance. Food intake was consistently higher in the SCP group throughout the experimental period (Figure 1K); food intake differences became statistically significant from Day 5 onward (*p* < 0.05 to *p* < 0.01). Similarly, water intake exhibited a parallel pattern (Figure 1L), with SCP-fed rats consuming significantly more water than controls across all time points, reflecting maintained metabolic activity and physiological homeostasis.

Comprehensive protein quality metrics for *R. eutropha* H16 SCP were listed in Figure 1M–P. True digestibility (TD) reached 85.73 ± 0.01%, indicating efficient enzymatic hydrolysis and absorption of the bacterial protein in the rat gastrointestinal tract. This high digestibility confirms that the protein is readily accessible for metabolic utilization despite the presence of bacterial cell wall components. However, the biological value (BV), representing the proportion of absorbed nitrogen retained for tissue synthesis, was moderate at 49.37 ± 0.03%. Correspondingly, net protein utilization (NPU), the product of TD and BV, was 42.33 ± 0.03%. These values suggest that while *R. eutropha* H16 SCP is efficiently digested and absorbed, a significant fraction of absorbed amino acids is catabolized rather than incorporated into body proteins, potentially due to amino acid imbalance (particularly the histidine limitation identified in Section 3.2 or metabolic conversion). The protein digestibility-corrected amino acid score (PDCAAS), a regulatory metric combining amino acid pattern with digestibility, was 0.71 (Table 2, Figure S2). This value was derived based on the score of the first limiting amino acid (histidine AAS = 0.83 ± 0.06) and adjusted for true digestibility (TD = 85.73%), resulting in a PDCAAS of approximately 0.71 for *R. eutropha* H16 SCP. This is comparable to that of plant proteins such as wheat (PDCAAS ≈ 0.68) but lower than that of high-quality animal proteins, including egg (PDCAAS ≈ 1.0) and casein (PDCAAS ≈ 1.0).

### 3.4. In Vitro Safety Assessment

#### 3.4.1. Chemical Contaminant Screening and Microbiological Safety Assessment

The concentrations of inorganic pollutants in *R. eutropha* H16 SCP are summarized in Table 3. Cadmium was present at <0.08 mg/kg, far below the FAO safe limit of 0.5 mg/kg [40] and the Codex Alimentarius maximum level of 0.2 mg/kg [52]. Chromium was detected at 2.08 mg/kg, remaining within the Chinese hygienic standard for feed of 5 mg/kg [40]. The total arsenic content was 0.183 mg/kg, substantially lower than the Codex Alimentarius limit of 0.5 mg/kg [52]. Lead was below 2 mg/kg, compliant with the maximum permissible concentration (MPC) of 3.0 mg/kg [41]; however, it should be noted that this value may marginally exceed the more stringent Codex Alimentarius limit of 0.3 mg/kg [52], warranting further optimization of upstream cultivation or downstream purification processes. Mercury was detected at <0.003 mg/kg, well below the MPC of 0.1 mg/kg [41] and the Codex Alimentarius limit of 0.1 mg/kg [52]. Fluorine was measured at 6 mg/kg, within the allowable range for feed ingredients (<35–40 mg/kg) [42]. Except for lead, which merits continued monitoring under the more conservative Codex threshold, all inorganic pollutants tested fell within their respective regulatory limits.

Aflatoxin B_1_ was below the detection threshold of 2 µg/kg, well within the maximum levels of 5 µg/kg established by both the Codex Alimentarius Commission and the European Commission [53] (Table 3). Microbiological assessment showed a total bacterial count of 2.5 × 10^4^ CFU/g, below the maximum permissible limit of 10^5^ CFU/g [44]. Neither *Salmonella* spp. nor *Shigella* spp. were detected in any of the samples tested (Table 3). Collectively, these results indicate that *R. eutropha* H16 SCP satisfies the applicable safety criteria for both chemical and microbiological contaminants.

#### 3.4.2. *In Vitro* Mammalian Chromosome Aberration Test

The *in vitro* mammalian chromosomal aberration assay was performed to evaluate the potential clastogenicity of *R. eutropha* H16 SCP. As shown in Table 4, no statistically significant increase (*p* > 0.05) in chromosomal aberration frequency was observed at any tested concentration (1.25, 2.5, and 5.0 mg/mL) compared with the negative control, regardless of metabolic activation status (±S9). The aberration rates across all *R. eutropha* H16 SCP-treated groups ranged from 0% to 1%, comparable to those of the corresponding negative controls (−S9: 0%; +S9: 0%). A constant count of 100 well-spread metaphase cells was maintained for analysis in each group, confirming the absence of overt cytotoxicity and adequate cell viability for aberration scoring. In contrast, the positive controls—methyl methanesulfonate (MMS, 50 µg/mL, −S9) and cyclophosphamide (CP, 10 µg/mL, +S9)—each elicited a significant increase in aberration frequency to 25% (*p* < 0.05), thereby validating the sensitivity and reliability of the test system. Collectively, these results indicate that *R. eutropha* H16 SCP tested negative in the *in vitro* chromosomal aberration assay and does not exhibit clastogenic potential under the conditions tested.

### 3.5. In Vivo Genotoxicity Assessment: Micronucleus Test

The *in vivo* mammalian erythrocyte micronucleus test was conducted to evaluate the genotoxic potential of the *R. eutropha* H16 single-cell protein by detecting chromosomal damage or interference with mitotic spindle apparatus function. As presented in Table 5, the micronucleus rates in bone marrow polychromatic erythrocytes (PCEs) of both male and female KM mice across all treatment groups (2.50, 5.00, and 10.00 g/kg BW) showed no statistically significant differences compared to the negative control group (*p* > 0.05). Specifically, the micronucleus rates in the treatment groups ranged from 2.0‰ to 3.5‰, which were comparable to those observed in the negative control group (3.2‰ for females and 3.3‰ for males). Furthermore, the PCE/NCE (polychromatic erythrocyte/normochromatic erythrocyte) ratios in all treatment groups remained stable at approximately 1.94–2.11, showing no significant deviation from the negative control values (*p* > 0.05). This indicates that *R. eutropha* H16 single-cell protein exerted no cytotoxic effects on the bone marrow hematopoietic system. In contrast, the positive control group treated with cyclophosphamide (CP, 0.04 g/kg BW) exhibited a markedly elevated micronucleus rate of 51.8‰ (females) and 55.0‰ (males), which was significantly higher than that of the negative control group (*p* < 0.01), thereby validating the sensitivity and reliability of the experimental system. Collectively, these results demonstrate that the *R. eutropha* H16 single-cell protein yielded negative results in the mammalian erythrocyte micronucleus test, indicating the absence of *in vivo* clastogenic or aneugenic activity.

### 3.6. Acute Oral Toxicity Test Results of R. eutropha H16 Single-Cell Protein in Mice and Rats

The acute oral toxicity of *R. eutropha* H16 single-cell protein was evaluated in KM mice and SD rats according to the National Food Safety Standard for Acute Oral Toxicity Test (GB 15193.3-2014) [47]. As presented in Table 6, no mortality was observed in either male or female mice in the treatment group administered the maximum feasible dose of 10,231.3 mg/kg and 10,171.1 mg/kg body weight (BW) throughout the 14-day observation period. The mortality rate was 0% for both sexes. During the experimental period, all animals in the treatment group exhibited normal physiological status, including good mental state, normal locomotor activity, glossy fur, and regular food and water consumption. No clinical signs of toxicity, abnormal behavior, or adverse reactions were observed. As presented in Figure S3, body weight measurements revealed a consistent and progressive increase in both the treatment and control groups, with no statistically significant differences between groups (*p* > 0.05). Based on these results, the median lethal dose (LD_50_) of *R. eutropha* H16 single-cell protein exceeded 10,000 mg/kg BW in both mice and rats. According to the UN Globally Harmonized System (GHS), substances with an oral LD_50_ greater than 5000 mg/kg in rats are generally not classified for acute toxicity, as they are considered to pose low acute hazard under normal conditions of use. The consistent absence of mortality and adverse effects across two rodent species provides robust evidence supporting the acute safety of *R. eutropha* H16 single-cell proteins for potential feed and food applications.

### 3.7. Subacute Toxicity Evaluation Results

**Figure 2:**
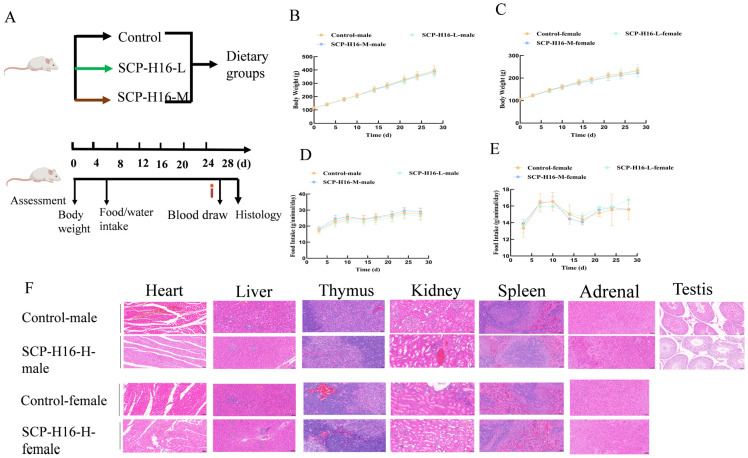
Subacute toxicity assessment of *R. eutropha* H16 SCP. (**A**) Schematic diagram of the subacute toxicity experimental design. (**B**) Body weight gain rate of male rats. (**C**) Body weight gain rate of female rats. (**D**) Food consumption growth rate of male rats. (**E**) Food consumption growth rate of female rats. SCP-H16-L, low-dose *R. eutropha* H16 SCP group (6.25% protein replacement); SCP-H16-M, medium-dose *R. eutropha* H16 SCP group (12.5% protein replacement); SCP-H16-H, high-dose *R. eutropha* H16 SCP group (25% protein replacement). (**F**) Representative histopathological images of heart, liver, spleen, kidney, thymus, testis, and adrenal gland from male and female rats in the control and high-dose groups. Magnification: 20× for all organs. SCP-H16-H, *R. eutropha* H16 SCP high-dose group.

A preliminary 28-day dose-ranging study compared a control diet with isonitrogenous, isocaloric SCP replacement at 25%, 50%, and 100% substitution levels. Overt organ-specific toxicity was observed even at the lowest substitution level (25%): Females exhibited a significantly decreased liver-to-body weight ratio (*p* < 0.05). Histopathology of high-dose females revealed hepatocellular steatosis, connective tissue proliferation, lymphocytic infiltration (Figure 2F), and renal tubular epithelial edema correlating with elevated serum uric acid, suggesting impaired tubular excretory function. Males showed only sporadic, mild renal tubular vacuolation without definitive structure–function disruption (Tables S5 and S6). No necrosis or fibrotic changes were detected in other organs. Accordingly, the dose range was narrowed to 6.25% (SCP-H16-L) and 12.5% (SCP-H16-M) to identify the no-observed-adverse-effect level (NOAEL).

At the revised doses, the low-dose group (6.25%) exhibited an entirely clean safety profile. All endpoints (body weight, food consumption, hematology, serum biochemistry, antioxidant markers, renal function, inflammatory cytokines, and organ-to-body weight ratios) remained within normal physiological ranges in both sexes, supporting its identification as safe *in vivo*. The medium-dose group (12.5%), by contrast, produced isolated, sex-specific deviations. In males, glutathione peroxidase (GPx) activity was significantly decreased (*p* < 0.01), serum creatinine (Cr) was reduced (*p* < 0.05), and serum albumin (ALB) levels were elevated (*p* < 0.05; Table S8); hematological parameters remained unremarkable. In females, no significant differences in hematology or serum biochemistry were observed relative to controls (Tables S7 and S8). Notably, ALB elevations observed in males of the medium-dose group (12.5%), combined with unchanged liver-to-body weight ratios (Table S9), suggested adaptive metabolic upregulation rather than hepatotoxicity. Overall, the 6.25% replacement level was established as the NOAEL. However, the mild deviations observed at the 12.5% level, though lacking histopathological confirmation, warrant further monitoring in longer-term studies.

## 4. Discussion

Global population growth by 2050 will substantially increase protein demand [54], yet traditional animal protein production entails considerable environmental and ethical costs [8,55]. Single-cell proteins (SCPs) from microbial fermentation offer a promising alternative owing to their high efficiency, land independence, and substrate versatility. This study systematically evaluated the nutritional quality and safety of *R. eutropha* H16 biomass to generate regulatory-grade evidence for advancing this SCP toward food and feed applications.

*R. eutropha* H16 SCP contains 71.87 ± 5.05 g protein/100 g dry weight, comparable to or exceeding soybean meal (approximately 44–48%) [56] and fishmeal (approximately 60–72%) [57]. Its AAS (0.83 ± 0.06), EAAI (1.46 ± 0.04), and essential amino acid composition closely approximate the FAO/WHO reference pattern [32]. The PER (2.41) in male rats surpassed wheat (0.8) and soybean (2.2), and the true digestibility (85.73%) confirmed efficient gastrointestinal absorption. However, BV (49.37%) and NPU (42.33%) were notably lower than TD, indicating limited post-absorptive retention for tissue synthesis. This is likely attributable to subtle amino acid imbalances, non-protein nitrogen compounds absorbed but not utilized for protein anabolism. In addition, bacterial cell wall components may partially interfere with amino acid utilization. Accordingly, the PDCAAS (0.71) positions this SCP above wheat protein but below casein, suggesting room for improvement through strain optimization or downstream processing.

A critical finding is the clear dose dependency of adverse effects (Tables S4–S6). At high doses (25–100%), overt toxicity included significant organ coefficient alterations, as well as hepatocellular steatosis with connective tissue proliferation and lymphocytic infiltration in females. Furthermore, renal tubular epithelial edema was noted, correlating with elevated serum uric acid consistent with nucleic acid-derived purine overload [58]. The medium-dose group (12.5%) exhibited subclinical, sex-specific perturbations: Females showed no significant differences in hematological or serum biochemical parameters relative to controls, whereas males displayed decreased GPx activity coupled with elevated ALB levels. Notably, these antioxidant alterations in males had no counterpart in females, suggesting sex-specific susceptibility that warrants further mechanistic investigation. Crucially, all adverse findings were confined to the medium- and high-dose groups. The low-dose group (6.25% protein replacement) showed no treatment-related abnormalities across any endpoint in either sex, supporting its designation as the NOAEL and providing a robust reference for establishing safe inclusion levels.

The acute oral LD_50_ exceeded 10,000 mg/kg BW in both mice and rats, well above the GHS 5000 mg/kg classification threshold, confirming negligible acute hazard. Genotoxicity assessments were uniformly negative: chromosomal aberration rates of 0–1% *in vitro* and no increase in micronucleus frequency *in vivo*, with stable PCE/NCE ratios ruling out bone marrow cytotoxicity. All monitored chemical contaminants (arsenic, lead, mercury, chromium, cadmium, fluorine, and aflatoxin B_1_) fell well below applicable national limits, and pathogenic microorganisms (*Salmonella*, *Shigella*) were undetected.

This work establishes a comprehensive evaluation framework integrating *in vitro* and *in vivo* methodologies for novel microbial protein assessment and contributes baseline data on bacterial protein metabolism in mammalian systems. The generated dataset, including nutritional characterization, acute and subacute toxicity, genotoxicity, and contaminant profiling, constitutes the core dossier for regulatory submission and potential catalog inclusion of *R. eutropha* H16 SCP. Future studies should extend to chronic and reproductive/developmental toxicity testing and explore strain engineering or processing strategies to improve BV and NPU.

## 5. Conclusions

This study provides comprehensive nutritional and safety evaluations of *Ralstonia eutropha* H16-derived single-cell proteins (SCPs), establishing its potential as a sustainable alternative protein source. *R. eutropha* H16 SCP exhibited exceptional protein content exceeding conventional plant proteins and comparable to premium fishmeal, with a high essential amino acid index. The protein efficiency ratio surpassed those of major plant proteins, and true digestibility confirmed efficient gastrointestinal utilization despite bacterial cell wall structures. However, moderate biological value, net protein utilization, and PDCAAS values indicate that post-absorptive retention remains below that of premium animal proteins, suggesting opportunities for improvement through strain optimization or blending with complementary sources. From a safety perspective, the multi-tiered assessment established a robust biosafety profile. All monitored heavy metals and mycotoxins were consistently below regulatory limits. Both *in vitro* chromosomal aberration and *in vivo* micronucleus tests yielded negative results, ruling out genotoxic potential. Acute oral LD_50_ values exceeded 10,000 mg/kg BW in both mice and rats, classifying this SCP as practically non-toxic. In the 28-day subacute toxicity study, adverse effects were confined to the medium- and high-dose groups, whereas the low-dose group (6.25% protein replacement) showed no treatment-related abnormalities, supporting its designation as the NOAEL.

## 6. Perspectives

As a novel food resource, the approval of *R. eutropha* H16 SCP for inclusion in feed or food ingredient catalogs requires adherence to stringent regulatory procedures, including submission of comprehensive safety, nutritional, and stability data. Simultaneously, enhancing awareness and acceptance of microbial proteins among stakeholders throughout the supply chain and among consumers represents an indispensable component of the commercialization process. Although acute toxicity and genotoxicity data support a low-risk profile, the current evaluation has primarily focused on acute and subacute toxicity. Future studies should include chronic toxicity investigations to fully characterize long-term safety. If human food applications are considered, additional assessments such as allergenicity evaluation, gut microbiota impact analysis, and human tolerance trials should be conducted in accordance with the novel food regulatory framework. In terms of nutrition, to address the limitation of “high digestibility but low utilization efficiency” of this SCP, fermentation process optimization or strain metabolic engineering should be employed to overcome histidine limitations and improve the composition of sulfur-containing amino acids, thereby enhancing *in vivo* retention efficiency. Concurrently, reducing the nucleic acid content of the biomass is necessary to decrease nitrogen excretion burden, which can be achieved through culture condition optimization or genetic editing. Integrating upstream strain engineering (e.g., enhancing sulfur-containing amino acid synthesis) with downstream processing (cell wall disruption and protein purification) can simultaneously improve nutritional quality while ensuring safety, laying a foundation for subsequent application research.

## Figures and Tables

**Table 1:** Amino acid composition of single-cell protein from *R. eutropha* H16 and other daily dietary proteins.

Amino Acids	Soy ^i^	Wheat ^i^	Casein ^i^	Pea ^i^	Corn ^i^	Potato ^i^	Egg ^i^	Whey ^i^	Milk ^i^	SCP-H16
**Essential Amino Acids**										
Threonine	2.30	1.80	2.60	2.50	1.80	4.10	2.00	5.40	3.50	3.04 ± 0.24
Methionine	0.30	0.70	1.60	0.30	1.10	1.30	1.40	1.80	2.10	1.37 ± 0.09
Phenylalanine	3.20	3.70	3.10	3.70	3.40	4.20	2.30	2.50	3.50	3.05 ± 0.41
Histidine	1.50	1.40	1.70	1.60	1.10	1.40	0.90	1.40	1.90	1.25 ± 0.10
Lysine	3.40	1.10	4.60	4.70	1.00	4.80	2.70	7.10	5.90	3.89 ± 0.32
Valine	2.20	2.30	3.00	2.70	2.10	3.70	2.00	3.50	3.60	3.91 ± 0.43
Isoleucine	1.90	2.00	2.30	2.30	1.70	3.10	1.60	3.80	2.90	2.75 ± 0.34
Leucine	5.00	5.00	5.80	5.70	8.80	6.70	3.60	8.60	7.00	5.12 ± 0.17
∑EAA	19.90	18.00	24.80	23.60	21.00	29.30	16.50	34.10	30.30	24.38 ± 0.51
**Non-Essential Amino Acids**										
Serine	3.40	3.50	3.40	3.60	2.90	3.40	3.30	4.00	4.00	2.34 ± 0.30
Glycine	2.70	2.40	1.20	2.80	1.60	3.20	1.40	1.50	1.50	3.94 ± 0.21
Glutamic acid	12.40	26.90	13.90	12.90	13.10	7.10	5.10	15.50	16.70	7.55 ± 0.51
Proline	3.30	8.80	6.50	3.10	5.20	3.30	1.80	4.80	7.30	2.82 ± 0.04
Cysteine	0.20	0.70	0.10	0.20	0.30	0.30	0.40	0.80	0.20	NA
Alanine	2.80	1.80	2.00	3.20	4.80	3.30	2.60	4.20	2.60	6.00 ± 0.07
Tyrosine	2.20	2.40	3.40	2.60	2.70	3.80	1.80	2.40	3.80	2.23 ± 0.29
Arginine	4.80	2.40	2.10	5.90	1.70	3.30	2.60	1.70	2.60	4.40 ± 0.18
∑NEAA	31.90	48.90	32.50	34.40	32.30	27.80	19.00	34.90	38.60	29.29 ± 0.74
∑TAA	51.80	66.90	57.30	58	53.30	57.10	35.50	69.00	68.90	53.67 ± 1.05
∑EAA/∑NEAA	0.62	0.37	0.76	0.67	0.65	1.05	0.87	0.98	0.78	0.83 ± 0.02
∑EAA/∑TAA	0.38	0.27	0.43	0.41	0.39	0.51	0.47	0.49	0.44	0.45 ± 0.01

SCP-H16: *R. eutropha* H16 SCP; NA: not tested; EAA: non-essential amino acids; NEAA; non-essential amino acids; TAA: total amino acid; i: data source [49].

**Table 2:** Amino acid score of single-cell protein from *R. eutropha* H16.

	Protein Scoring Standards		SCP-H16	
	Egg (g/100 g) ^j^	FAO/WHO (g/100 g) ^k^	Content (g/100 g)	AAS
Histidine	0.90	1.50	1.25 ± 0.10	0.83 ± 0.06
Threonine	2.00	2.30	3.04 ± 0.24	1.32 ± 0.11
Valine	2.00	3.90	3.91 ± 0.43	1.00 ± 0.11
Cysteine	0.40	0.60	/	/
Methionine	1.40	1.60	1.37 ± 0.09	0.85 ± 0.06
Methionine + Cysteine	1.80	2.20	1.37 ± 0.09	0.62 ± 0.04
Isoleucine	1.60	3.00	2.75 ± 0.34	0.92 ± 0.11
Leucine	3.60	5.90	5.12 ± 0.17	0.87 ± 0.03
Phenylalanine + Tyrosine	4.10	3.80	5.29 ± 0.67	1.39 ± 0.18
Lysine	2.70	4.50	3.89 ± 0.32	0.87 ± 0.07
∑EAA	16.50			/
EAAI	1.46 ± 0.04			
PDCAAS	0.71			

SCP-H16: *R. eutropha* H16 SCP; AAS: amino acid score; sources: j [49], k [50], and FAO/WHO requirements in g/100 g of proteins for an ideal adult protein.

**Table 3:** Screening for illegal additives.

Items	Testing Metrics	Results
Inorganic Pollutant	Arsenic	0.183 mg/kg
	Lead	<2 mg/kg
	Mercury	<0.003 mg/kg
	Chromium	2.08 mg/kg
	Cadmium	<0.08 mg/kg
	Fluoride	6 mg/kg
Mycotoxin	Aflatoxin B1	<2 μg/kg
	Coagulase-positive (*Staphylococcus*)	<10
Pathogen	*Shigella*	NA
	*Salmonella*	NA
	Total bacterial count	2.5 × 10^4^ CFU/g

NA: Not detected.

**Table 4:** *In vitro* mammalian chromosome aberration assessment results of *R. eutropha* H16 SCP.

Groups	Final Concentration	Observe Cells/Piece		Aberrant Cells/Piece		Distortion Rate/%	
		+S9	−S9	+S9	−S9	+S9	−S9
Negative controls	/	100	100	1	0	1	0
Positive controls	50 μg/mL	100	100	/	29	/	25 *
	10 µg/mL	100	100	26	/	25 *	/
Test object	5 mg/mL	100	100	1	4	1	1
	2.5 mg/mL	100	100	0	0	0	0
	1.25 mg/mL	100	100	2	0	1	0

S9: supernatant fraction obtained at 9000× *g*; * significantly different from the negative control group, *p* < 0.05.

**Table 5:** *In vivo* mammalian erythrocyte micronucleus assessment results of *R. eutropha* H16 SCP.

Dose (g/kg BW)	Sex	Micronucleus Count (n)	Micronucleus Rate (‰)	PCE/NCE Ratio
Negative Control	Female	32	3.2 ± 1.35	1.99 ± 0.14
	Male	33	3.3 ± 1.48	1.99 ± 0.15
Low Dose (2.50)	Female	21	2.1 ± 0.82	1.94 ± 0.07
	Male	23	2.3 ± 0.27	2.11 ± 0.16
Medium Dose (5.00)	Female	35	3.5 ± 0.29	2.07 ± 0.13
	Male	20	2.0 ± 0.35	2.06 ± 0.17
High Dose (10.00)	Female	25	2.5 ± 0.35	2.0 ± 0.17
	Male	34	3.4 ± 1.39	2.11 ± 0.12
Positive Control (CP, 0.04)	Female	518	51.8 ± 3.29 **	—
	Male	550	55.0 ± 7.34 **	—

Values are expressed as mean ± SD (n = 5 per sex per group). ** Highly significantly different from the negative control group, *p* < 0.01; CP: cyclophosphamide; PCE: polychromatic erythrocytes; NCE: normochromatic erythrocytes.

**Table 6:** Acute oral toxicity test results of *R. eutropha* H16 single-cell protein in KM mice and SD rats.

	Actual Dose of Poison (mg/kg·BW)	No. Animals	General Health	Symptoms of Poisoning in Animals	
Group			No. anomalies	No. Reactions	No. deaths
KM Mice	10,231.3	10	0	0	0
SD Rat	10,171.1	10	0	0	0

## Data Availability

The original contributions presented in this study are included in the article/Supplementary Materials. Further inquiries can be directed to the corresponding authors.

## Supplementary Materials

The following supporting information can be downloaded at https://www.mdpi.com/article/10.3390/foods15101813/s1, Table S1. The composition of the experimental diets (g/1000 g). Table S2. The composition of the experimental diets (g/1000 g). Table S3. Nutritional Components of Single-Cell Protein from *R. eutropha* H16. Table S4. The first batch of organ coefficients of subacute toxicity. Table S5. The first batch of serological biochemical analysis results of subacute toxicity. Table S6. The first batch of hematological analysis results of subacute toxicity. Table S7. The second batch of hematological analysis results of subacute toxicity. Table S8. The second batch of serological biochemical analysis results of subacute toxicity. Table S9. The second batch of organ coefficients of subacute toxicity. Figure S1. Comparison of the protein efficiency ratio. Figure S2. Comparison of different PDCAAS, BV, and NPU, TD. Figure S3. Acute toxicity—14-day weight change. References [59,60,61,62,63,64,65,66,67,68] are cited in the Supplementary Materials.
