# Supplementary material for: Nutritional Value and Food Safety Assessment of Single-Cell Protein Derived from Ralstonia eutropha for Food Applications
Source: Foods. 2026 May 20;15(10):1813. doi: 10.3390/foods15101813 (PMC13205168; doi:10.3390/foods15101813)
Supplement: Supplementary file 1 [file foods-15-01813-s001.zip › foods-4254892-supplementary.pdf]

Supplementary Information (SI)

Nutritional Value and Food Safety Assessment of Novel Single-Cell Protein Derived from *Ralstonia eutropha* for Food applications

This file includes:

A. **Supplementary tables**

Tables S1 to S9

Table S1. The composition of the experimental diets (g/1000g).

SCP Diet (g)			Control (g)		
Ingredient	Proportion (g)	Energy (kcal)	Ingredient	Proportion (g)	Energy (kcal)
SCP (H16)	148.20	430.97	Casein	104.00	416.00
L-Cystine	0.00	0.00	L-Cystine	1.50	6.00
Corn starch	464.70	1858.80	Corn starch	495.00	1980.00
Maltodextrin	132.00	528.00	Maltodextrin	132.00	528.00
Sucrose	100.00	400.00	Sucrose	100.00	400.00
Cellulose	40.50		Cellulose	50.00	
Soybean oil	67.10	603.90	Soybean oil	70.00	630.00
Mineral mix	35.00		Mineral mix	35.00	
Vitamin mix	10.00	40.00	Vitamin mix	10.00	40.00
Choline	2.50		Choline	2.50	
TBHQ	0.01		TBHQ	0.01	
Total	1000.00	3861.67	Total	1000.00	4000.00
**Macronutrient Ratio**	**Mass**	**Energy**	**Macronutrient Ratio**	**Mass**	**Energy**
Protein	0.10	0.10	Protein	0.10	0.11
Fat	0.07	0.16	Fat	0.07	0.16
Carbohydrate	0.71	0.73	Carbohydrate	0.74	0.74

Where SCP (H16): *R. eutropha* H16 SCP.

Table S2. The composition of the experimental diets (g/1000g).

SCP Diet (g)			Protein-free Control (g)		
Ingredient	Proportion (g)	Energy (kcal)	Ingredient	Proportion (g)	Energy (kcal)
SCP (H16)	148.20	430.97	Casein	0.00	0.00
L-Cystine	0.00	0.00	L-Cystine	0.00	0.00
Corn starch	464.70	1858.80	Corn starch	600.50	2402.00
Maltodextrin	132.00	528.00	Maltodextrin	132.00	528.00
Sucrose	100.00	400.00	Sucrose	100.00	400.00
Cellulose	40.50		Cellulose	50.00	
Soybean oil	67.10	603.90	Soybean oil	70.00	630.00
Mineral mix	35.00		Mineral mix	35.00	
Vitamin mix	10.00	40.00	Vitamin mix	10.00	40.00
Choline	2.50		Choline	2.50	
TBHQ	0.01		TBHQ	0.01	
Total	1000.00	3861.67	Total	1000.00	4000.00
**Macronutrient Ratio**	**Mass**	**Energy**	**Macronutrient Ratio**	**Mass**	**Energy**
Protein	0.10	0.10	Protein	0.00	0.00
Fat	0.07	0.16	Fat	0.07	0.16
Carbohydrate	0.71	0.73	Carbohydrate	0.84	0.84

Where SCP (H16): *R. eutropha* H16 SCP.

Table S3. Nutritional Components of Single-Cell Protein from *R. eutropha* H16.

Sample	Protein	Fat	Moisture	Ash	Dietary Fiber
SCP-H16	71.87±5.05	2.00±2.17	2.48±1.38	4.87±0.95	NA
Arthrospira maxima[a]	62.81-70.24	5.97-7.30	NR	NR	NR
Chlorella pyrenoidosa[b]	45.02	22.89	6.42	4.56	NR
Rhodopseudomonas palustris[c]	65	3	10	14	NR
Methylophilus methylotrophus[d]	81.3	7.2	NR	9.1	NR
Methylococcus capsulatus[e]	69	0.17	9.5	14.4	NR
Brewer's spent yeast[f]	64.1	1.32	7.7	14	NR
Saccharomyces cerevisiae[g]	47.78	2.36	3.75	7.85	3.38
Candida utilis[h]	54.5	0.1	NR	11.5	14.2

Where SCP-H16: R. eutropha H16 SCP; NR: values not shown were not reported in the respective experiment; NA: not detected; sources: a [59], b [60], c [61], d [62], e [63], f [64], g [65], h [66].

Table S4. The first batch of organ coefficients of subacute toxicity.

Parameters	con-male	25%-male	50%-male	100%-male	con-female	25%-female	50%-female	100%-female
Heart	0.33±0.03	0.36±0.04	0.34±0.02	0.36±0.02	0.34±0.03	0.35±0.03	0.33±0.03	0.34±0.02
Thymus	0.22±0.03	0.21±0.04	0.2±0.02	0.2±0.04	0.22±0.04	0.21±0.03	0.22±0.04	0.22±0.02
Liver	3.86±0.26	3.7±0.57	3.55±0.3	3.42±0.3	3.85±0.37	3.53±0.23*	3.49±0.17*	3.57±0.22
adrenal	0.023±0	0.022±0	0.024±0	0.024±0	0.033±0	0.029±0	0.030±0	0.030±0
Spleen	0.21±0.02	0.24±0.06	0.23±0.05	0.19±0.02	0.24±0.04	0.26±0.04	0.25±0.04	0.24±0.04
Kidney	0.97±0.05	0.97±0.08	0.96±0.03	0.99±0.06	0.91±0.08	0.92±0.06	0.97±0.07	1.15±0.15***
Testis	1.03±0.08	1.07±0.09	1.05±0.08	1.44±0.07***	/	/	/	/

The results are expressed as mean ± standard deviation (n=10 for male/female). *Significant difference compared with the control group, $p < 0.05$; **Highly significant difference compared with the control group, $p < 0.01$; ***Extremely significant difference compared with the control group, $p < 0.001$.

Table S5. The first batch of serological biochemical analysis results of subacute toxicity.

**Parameters**	**con-male**	**25%-male**	**50%-male**	**100%-male**	**con-female**	**25%-female**	**50%-female**	**100%-female**
CRP(ng/mL)	399.46±45.04	356.33±37.94	452.62±44.87	516.55±62.83*	**502.61±43.6	346.53±37.84*	**407.91±45.12***	473.16±48.5
GGT(U/L)	22.84±14.3	10.56±1.23	10.48±1.23	18.47±29.37	10.27±1.6	9.53±0.46	9.61±0.79	9±0.54*
ALB(g/L)	30.54±1.31	30.66±1.14	30.34±0.76	26.45±1.22***	35±1.03	33.99±2.93	33.44±2.42	30.24±1.49***
IL-6（pg/mL）	28.03±3.4	26.07±2.3	32.76±3.46*	39.29±3.53***	36.18±2.19	26.96±1.84***	30.34±3.12***	34.64±2.78
ALT(U/L)	37.29±6.74	34.23±8.82	41.4±9.4	56.1±8.64***	33.19±7.31	28.69±6.35	28.29±4.65	38±8.41
SOD(U/mL)	14.99±5.21	20.37±6.02*	10.38±2.44	7.32±1.86***	8.42±2.36	20.57±4.05***	17.95±4.13***	10.76±2.69
TG(mmol/L)	0.53±0.1	0.8±0.22	1.26±0.39***	1.76±0.39***	0.48±0.16	0.56±0.16	0.66±0.24	0.92±0.31***
GSH-Px(nmol /min/mL)	596.92±18.23	609.04±16.38	578.96±18.28	537.07±29.28*	**541.17±22.27	607.69±19.13*	**574.27±17.02**	552.16±25.94
Cr(umol/L)	110.77±69.28	34.76±4.66***	30.56±3.18***	33.62±4.85***	43.94±27.45	35.46±3.26	35.2±3.38	37.4±4.53
AKP(U/L)	128.05±45	128.78±28.33	155.47±24.61	187.05±60.46*	66.35±18.07	75.7±26.62	91.13±21.82	112.12±35.5**
UA(umol/L)	108.56±52.1	101.9±20.81	94.91±10.02	84.35±10.95	107.99±31.76	99.34±12.56	92.65±10.25	86.83±10.11
AST(U/L)	105.08±16.05	97.1 ±12.9	111.48±18.69	132.92±12.44*	**120.9±18.33	98.8±16.01*	107.39±15.19	113.15±16.32
HGB(g/L)	58.2±5.72	60.68±4.32	55.77±5.41	46.97±6.41***	45.41±6.42	62±6.35***	58.43±5.5***	49.93±6.03
TC(mmol/L)	1.65±0.43	1.84±0.27	2.01±0.4	2.26±0.27**	2.2±0.26	2.11±0.52	1.89±0.3	1.72±0.32*

The results are expressed as mean ± standard deviation (n=10 for male/female). *Significant difference compared with the control group, $p < 0.05$; **Highly significant difference compared with the control group, $p < 0.01$; ***Extremely significant difference compared with the control group, $p < 0.001$.

Table S6. The first batch of hematological analysis results of subacute toxicity.

Parameters	con-male	25%-male	50%-male	100%-male	con-female	25%-female	50%-female	100%-female
WBC (10^9/L)	3.33±0.8	4.27±2.53	4.7±1.24	4.21±1.8	2.36±1.89	2.67±1.04	2.72±1.7	4.14±2.36
RBC (10^{12}/L)	6.69±0.25	6.14±2.18	7.05±0.36	6.69±2.36	5.94±3.01	7.12±0.41	6.59±2.34	6.48±2.31
HGB (g/L)	137.1±6.35	124.1±43.52	141±6.24	126.1±44.72	115±58.35	136.8±6.89	126.5±44.87	121.5±43.63
HCT (%)	39.01±1.97	35.61±12.52	40.68±2.05	36.65±12.99	32.22±16.29	38.9±1.91	35.58±12.62	34.92±12.48
MCV (fL)	58.28±1.24	57.31±3.18	57.71±1.39	54.33±2.31**	58.93±14.51	54.68±1.18	48.58±17.1	58.48±14.61
MCH (pg)	20.48±0.4	20.73±1.66	20±0.44	16.98±5.99	17.42±6.15	19.23±0.49	17.27±6.08	16.88±5.94
MCHC (g/dL)	35.16±0.22	36.36±4.8	34.67±0.35	30.99±10.91	32.01±11.26	35.18±0.54	35.54±0.33	31.32±11.02
PLT (10^9/L)	981.8±272.79	960.9±344.76	951.5±322.04	823.3±407.35	778.7±408.36	1025.7±310.27	824.9±332.18	891.8±380.58
MPV (fL)	7.55±0.25	7.36±0.17	7.48±0.32	7.28±0.22	7.54±0.36	7.45±0.16	7.36±0.19	7.24±0.14*
NEUT (10^3/μL)	0.55±0.09	1.24±1.21	1.25±0.51	1.61±0.84*	0.35±0.21	0.62±0.51	0.62±0.34	2.18±1.81**
LYMPH (10^3/μL)	2.5±0.65	3.08±1.29	3.06±1.03	2.71±0.38	2.1±1.57	1.84±0.56	2.15±1.19	1.98±0.7
MONO (10^3/μL)	0.24±0.1	0.28±0.17	0.34±0.08	0.3±0.1	0.12±0.05	0.16±0.06	0.2±0.11	0.28±0.16*
EO (10^3/μL)	0.04±0.02	0.06±0.03	0.04±0.02	0.04±0.01	0.04±0.04	0.05±0.02	0.06±0.03	0.05±0.03
BASO (10^3/μL)	0.01±0.01	0.01±0.01	0.01±0.01	0.01±0.01	0.01±0.01	0±0	0.01±0.01	0±0.01
NEUT% (%)	16.84±2.62	25.06±13.81	26.92±10.15	32.69±9.96**	16.11±8.18	20.99±8.49	20.81±5.3	42.63±20.49***
LYMPH% (%)	74.6±2.59	67.63±14.01	64.81±10.57	60.01±10.32*	76.7±8.33	71.05±8.63	70.2±4.44	49.52±19.01***
MONO% (%)	7.22±2.22	5.96±1.23	7.28±1.37	6.29±1.19	5.28±1.51	6.07±1.53	6.86±2.07	6.46±2.34
EO% (%)	1.13±0.59	1.24±0.43	0.85±0.23	0.89±0.36	1.44±0.75	1.8±0.68	1.95±0.28	1.29±0.51
BASO% (%)	0.21±0.17	0.11±0.15	0.14±0.13	0.12±0.12	0.47±1	0.09±0.15	0.18±0.24	0.1±0.15

All data are expressed as mean ± standard deviation (male/female, n = 10 per group). Statistical significance relative to the control group is indicated as follows: *$p < 0.05$, **$p < 0.01$, ***$p < 0.001$.

Table S7. The second batch of hematological analysis results of subacute toxicity.

**Parameters**	**Control-Male**	**SCP-H16-L-Male**	**SCP-H16-M-Male**	**Control-Female**	**SCP-H16-L-Female**	**SCP-H16-M-Female**
WBC (10^9/L)	4.59±0.76	4.69±2.13	5.17±1.47	4.7±2.3	3.83±1.71	2.82±1.33
RBC (10^{12}/L)	6.69±0.83	7.09±0.35	6.99±0.4	7.12±0.7	7.23±0.44	7.34±0.57
HGB (g/L)	134.6±15.33	139.56±8.53	140±5.72	142.8±13.36	142.3±5.95	144.56±8.97
HCT (%)	39.23±4.24	40.6±2	40.57±1.15	40.28±3.77	40.45±1.54	41.12±2.89
PLT (10^{12}/L)	1.02±0.12	1.1±0.09	8.03±20.99	0.82±0.45	1.05±0.23	0.73±0.43
NEUT (10^3/μL)	0.76±0.14	0.76±0.27	0.71±0.14	0.33±0.11	0.35±0.19	0.2±0.11
LYMPH (10^3/μL)	3.56±0.74	3.61±1.86	4.15±1.28	4.11±2.16	3.25±1.52	2.47±1.2
MONO (10^3/μL)	0.24±0.1	0.27±0.09	0.27±0.12	0.2±0.07	0.18±0.06	0.12±0.06
EO (10^3/μL)	0.03±0.01	0.04±0.02	0.03±0.01	0.06±0.04	0.04±0.02	0.03±0.01
BASO (10^3/μL)	0.01±0.01	0.01±0.01	0.01±0.01	0.01±0	0.01±0.01	0.01±0.01

All data are expressed as mean ± standard deviation (male/female, n = 10 per group). Statistical significance relative to the control group is indicated as follows: *p < 0.05, **p < 0.01.

Table S8. The second batch of serological biochemical analysis results of subacute toxicity.

Parameters	con-male	SCP-H16-L-male	SCP-H16-M-male	con-female	SCP-H16-L-female	SCP-H16-M-female
CRP(ng/mL)	466.24±58.85	464.98±91.23	424.33±78.83	453.34±72.72	471.18±60.54	483.1±49.57
GGT(U/L)	4.12±4.97	2.35±1.87	2.41±3.22	0.67±0.54	0.58±0.58	0.56±0.63
ALB(g/L)	30.04±1.21	31.54±2.16	32.01±1.65*	33.49±1.82	33.91±2.43	34.31±2.14
IL-6（pg/mL）	29.59±5.8	26.56±4.51	34.47±4.69	32.48±4.15	31.54±4.63	35.09±4.97
ALT(U/L)	29.12±6.61	25.93±6.08	23.82±5.73	23.48±2.83	19.63±4.56	22.49±8.77
SOD(U/mL)	18.99±6.04	15.01±3.34	14.44±2.75	12.74±3.98	12.94±3.63	9.04±2.9
TG(mmol/L)	1.1±0.68	0.92±0.21	0.84±0.25	0.67±0.28	0.49±0.25	0.55±0.27
GSH-Px(nmol /min/mL)	547.1±17.83	523.04±26.54	511.42±20.73**	503.93±30.99	507.35±28.54	511.69±23.91
Cr(umol/L)	78.14±59.9	46.29±45.23	28.54±4.79*	34.3±3.88	35.35±3.79	34.68±4.74
AKP(U/L)	120.92±27.92	152.21±29.81	152.78±37.21	84.05±18.25	88.5±21.93	97.86±19.47
UA(umol/L)	66.14±29.3	67.71±14.7	133.89±103*	84.92±11.87	82.73±10.72	94.06±19.74
AST(U/L)	81.38±15.68	84.94±18.96	86.99±42.38	90.27±26.63	89.38±15.92	122.65±86.1
TC(mmol/L)	1.46±0.35	1.49±0.42	1.6±0.31	1.98±0.27	1.76±0.3	1.84±0.41

All data are expressed as mean ± standard deviation (male/female, n = 10 per group). Statistical significance relative to the control group is indicated as follows: *$p < 0.05$, **$p < 0.01$, and ***$p < 0.001$.

Table S9. The second batch of of organ coefficients of subacute toxicity.

Parameters	Con-Male	SCP-H16-L-Male	SCP-H16-M-Male	Con-Female	SCP-H16-L-Female	SCP-H16-M-Female
Heart	0.33±0.03	0.34±0.02	0.33±0.02	0.36±0.02	0.35±0.03	0.37±0.02
Thymus	0.23±0.04	0.25±0.04	0.23±0.03	0.27±0.04	0.27±0.05	0.29±0.03
Liver	3.62±0.22	3.47±0.3	3.55±0.21	3.22±0.25	3.22±0.28	3.13±0.21
adrenal	0.018±0	0.019±0	0.019±0	0.031±0	0.030±0	0.030±0
Spleen	0.2±0.03	0.19±0.02	0.2±0.02	0.22±0.03	0.24±0.02	0.22±0.02
Kidney	0.85±0.05	0.92±0.08	0.89±0.04	0.89±0.06	0.92±0.06	0.87±0.06
Testis	0.81±0.09	0.84±0.07	0.81±0.05	-	-	-

The results are expressed as mean ± standard deviation (n=10 for male/female).

B. Supplementary figures

Figures S1 to S3

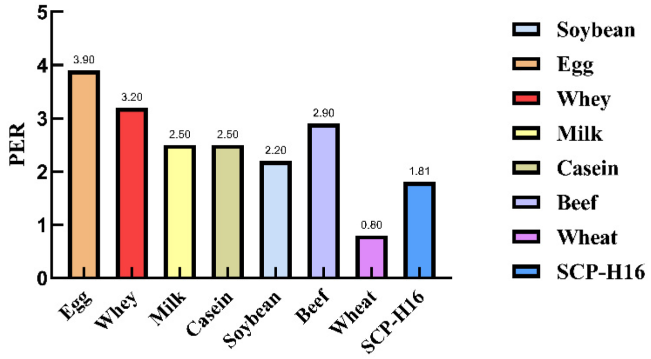

Figure S1. Comparison of the protein efficiency ratio.

Where data reference [67], SCP-H16: *R. eutropha* H16 SCP.

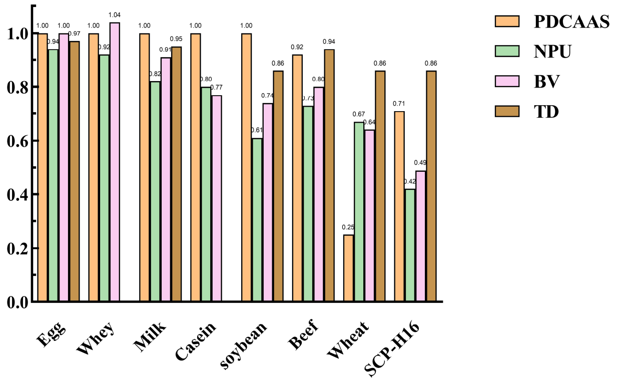

Figure S2. Comparison of different PDCAAS, BV, and NPU, TD.

Where data reference [68]; SCP-H16: *R. eutropha* H16 SCP.

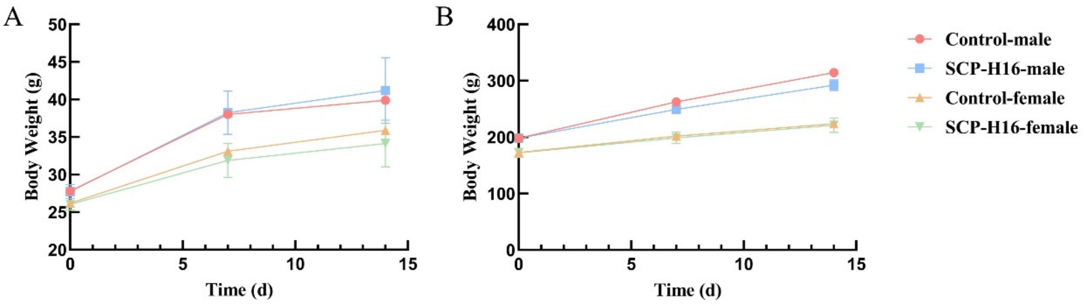

Figure S3. Acute toxicity - 14-day weight change.
